# A comparison of symptoms in older hospitalised cancer and non-cancer patients in need of palliative care: a secondary analysis of two cross-sectional studies

**DOI:** 10.1186/s12877-018-0721-7

**Published:** 2018-02-05

**Authors:** Aurélie Van Lancker, Ann Van Hecke, Sofie Verhaeghe, Matthias Mattheeuws, Dimitri Beeckman

**Affiliations:** University Centre for Nursing and Midwifery, Department of Public Health, Faculty of Medicine and Health Sciences, Ghent University, Ghent, Belgium

**Keywords:** Geriatric, Aged, Symptoms, Palliative care, Cancer, Non-cancer

## Abstract

**Background:**

Evidence on the differences in symptom patterns between older palliative cancer and non-cancer patients is lacking. The purpose of the study was to determine the differences in symptoms between older hospitalised palliative cancer and non-cancer patients.

**Methods:**

A secondary analysis of two multi-centre cross-sectional studies was performed. A validated instrument was used to assess the frequency and intensity of 40 symptoms in older hospitalised palliative cancer patients (*n* = 100) and older palliative non-cancer patients (*n* = 100). The data were collected between March 2013 and June 2015. Differences between groups were measured statistically.

**Results:**

Overall, similarities in symptom patterns were observed between cancer and non-cancer patients. Some minor differences were detected between the groups. Non-cancer patients experienced significantly more physical symptoms and functional dependence than cancer patients. Patients with cancer experienced higher levels of frequency and intensity of psychological symptoms compared to non-cancer patients.

**Conclusions:**

Healthcare professionals should be aware of the high occurrence of symptoms in both cancer and non-cancer patients, and they should be educated about the systematic assessment of symptoms in multiple domains by accounting for the occurrence of generic symptoms and disease-specific symptoms.

## Background

In palliative care, which aims to improve the quality of life of patients facing multiple symptoms related to a life-threatening illness and that of their family, particular emphasis is placed on the assessment and management of symptoms [1]. For some time, palliative care has focused on the care of patients with cancer [2]. Currently, more attention is devoted to introducing palliative care early in the trajectory of patients facing chronic illnesses other than cancer [2]. However, the referral of these patients to palliative care remains difficult because of the unpredictable course of chronic illness and the lack of adequate education on palliative care in caregivers [3].

Two systematic reviews have examined the occurrence of symptoms in patients with advanced cancer and other chronic illnesses [4, 5]. The authors of both reviews observed that cancer and non-cancer patients experienced multiple symptoms, with some commonalities in symptom patterns between the two groups [4, 5]. However, these conclusions were based on their interpretation of the data rather than on the statistical measurement of a difference between groups. Moreover, the authors concluded that most studies focused on physical symptoms and that only a few studies investigated social and existential symptoms. Additionally, Moens, Higginson, & Harding (2014) noted that more research is needed to compare symptoms in cancer and non-cancer patients using the same instrument and time point [4].

The available evidence focuses on adults in general [4, 5]. The results of these studies cannot be generalised to older populations (patients aged 65 years and older) due to the changes in physiology related to the ageing process and the higher prevalence of multimorbidity in this population [6]. Consequently, older patients are more vulnerable to experiencing a complex interplay of multiple problems and symptoms in different domains, concerning not only the physical domain but also the psychological, social, existential, and functional domains [7].

To our knowledge, there is a lack of evidence on the differences in symptom patterns between older palliative cancer and non-cancer patients. In accordance with Solano et al. (2006) and Moens et al. (2014), our hypothesis is that there is no difference in the experience of symptoms by older cancer versus non-cancer patients. This might strongly suggest that the need for palliative care should be need-based rather than diagnosis-based. In the last years, a need-based referral is given more attention, but remains difficult in clinical care.

## Methods

### Aim

The aim of this study was to determine the differences in (i) the number of symptoms per patient group and (ii) the frequency and intensity of symptoms between hospitalised older palliative cancer and non-cancer patients.

### Design

A secondary analysis of two multi-centre cross-sectional studies was performed. The first study focused on the frequency and intensity of symptoms and the treatments administered in hospitalised older patients with cancer and need of palliative care [8]. The second study was part of a study that focused on the frequency and intensity of symptoms in hospitalised older patients without cancer in need of palliative care [9].

### Setting and participants

The patients were recruited from geriatric and internal medicine wards of nine acute care hospitals (one teaching hospital and eight general hospitals) in Belgium. Patients were not recruited from the palliative care wards because in the Belgian healthcare system these specific patients need to have limited life expectancy of a few weeks to 3 months.

A convenience sample of older palliative cancer and non-cancer patients was invited to participate. Participants were eligible if they (i) were ≥65 years, (ii) were diagnosed with cancer, heart failure, obstructive lung disease, renal failure, or liver failure, (iii) were in the palliative phase of their disease, (iv) were able to communicate with the researcher, and (v) were able to provide written informed consent. Patients in the terminal phase of their disease were excluded. Patients in need of palliative care and patients in the terminal phase of their disease were defined by a set of criteria developed by a panel of experts (*n* = 7) with clinical expertise in oncology, palliative care and/or geriatric care. The criteria were based on definitions reported in the literature [1, 10–13]. This resulted in the following criteria for patients in need of palliative care: “Patients suffering from an incurable disease based on treatment options, general condition of the patient, multipathology and patient preferences. In contrast to healing, stabilisation of the disease is possible.” For patients in the terminal phase, the following criteria were used: “The last phase of life, as characterised by global organ failure that is clinically apparent in physical (e.g., respiratory) and mental (e.g., agitation) changes.”

### Instruments

#### Symptoms in cancer patients

**Fig. 1:**
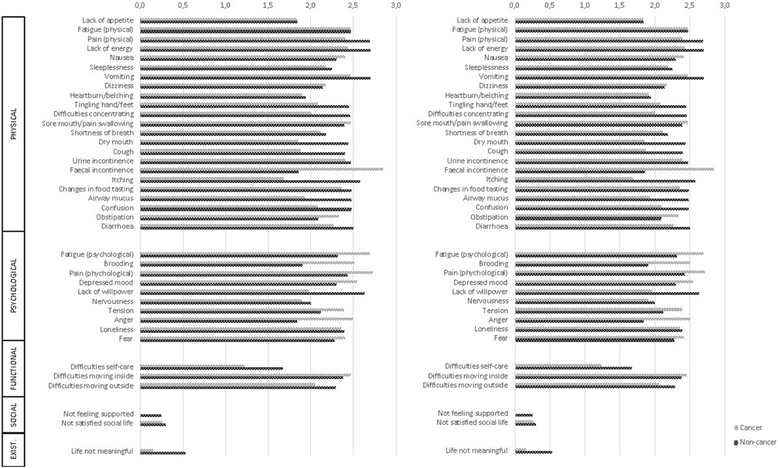
Frequency and Intensity of Symptoms in Older Cancer and Non-Cancer Patients. Mean values are presented. For frequency values ranged from 0 to 4, with 4 indicating always present. For intensity, values ranged from 0 to 3, with 3 indicating high intensity

The frequency and intensity of symptoms were collected using the Assessment Symptoms Palliative Elderly (ASPE) [14]. This instrument was developed to assess the frequency and intensity of symptoms in older palliative cancer patients. The instrument has shown good validity and reliability. In total, 40 symptoms (Fig. 1), including 24 physical, 10 psychological, and 3 functional symptoms and 2 items in the social and 1 item in the spiritual domain, were measured on (i) a 5-point Likert scale to assess frequency (0 = *never*; 1 = *rarely*; 2 = *sometimes*; 3 = *often*; 4 = *always*) and (ii) a 4-point Likert scale to assess intensity (0 = *not*; 1 = *somewhat*; 2 = *moderate*; 3 = *very serious*). The intensity of symptoms was assessed if the patient scored the frequency of the symptom as a 1 to 4. The ‘weight loss’ item had a dichotomised answer category (‘yes’ or ‘no’).

#### Symptoms in non-cancer patients

An adapted version of the ASPE was used. Based on a literature review of instruments developed to assess the symptoms of adults with heart failure, obstructive lung disease, renal failure and liver failure, eight items were added to the ASPE to increase the content validity of the instrument for non-cancer patients. These eight additional items were not included in the comparison analysis, because these items were not collected in the cancer group.

#### Demographic and clinical variables

Demographic and clinical variables were collected using a standardised form. The demographic variables were as follows: age, gender, living status, and living arrangements. The clinical variables included the following: type of illness for which the patient was considered in need of palliative care and the Flemish Triage Risk Screening Tool (fTRST) [15]. The fTRST is a screening tool which includes five items scored as present or absent [15]. The total score ranges between 0 and 5, in which a score ≥ 2 indicates having a geriatric risk profile [15].

### Data collection

In the period between March 2013 and June 2015, the researchers contacted the participating wards weekly to identify eligible patients. The responsible physician of the participating wards classified the patients as being in need of palliative care, in a terminal phase, or none of the previous two. After that a researcher approached the patients who met the inclusion criteria. Two researchers collected data on the frequency and intensity of symptoms. One researcher (AVL) collected data from the cancer patients, whereas the other researcher (MM) collected data from the non-cancer patients. The researchers completed the ASPE, the demographic variables and the fTRST based on a structured interview with the patient. The instructions and guidance provided by the researchers were reduced to a minimum and were standardised to improve the validity of the results. The first author (AVL), who developed the instrument and was therefore familiar with its use, explained the application of the instrument to the second researcher (MM) to optimise consistency in data collection. Information on the type of illness was collected through the electronic medical and paramedical records of the patients or was obtained from the responsible medical doctor or nurse.

### Ethical approval

Ethical approval was obtained from the ethics review committee of the teaching hospital (University Hospital Ghent) and general hospitals (Maria Middelares Hospital Ghent, General Hospital Sint-Lucas Ghent, Onze-Lieve-Vrouw van Lourdes Hospital Waregem, General Hospital Sint-Lucas Bruges, General Hospital Alma Eeklo, Onze Lieve Vrouw Hospital Alost, General Hospital Nikolaas, Sint-Andries Hospital Tielt) (B670201317036 and B670201523233). The participants received oral information about the aim of the study from a healthcare professional within the hospital. After preliminary consent, the researcher approached the participant to provide more detailed information about both the aim of the study and the participant’s anonymity. Written informed consent was obtained from all participants.

### Data analysis

Descriptive data were reported as the mean values with a standard deviation for normally distributed continuous variables, median values with 25th – 75th interquartile ranges for ordinal variables, and percentages for nominal variables. To improve the interpretation of the results, mean values with a standard deviation were also provided for the comparison of the frequency and intensity of symptoms between groups (cancer and non-cancer patients).

In the study in older palliative cancer patients, 400 patients participated. In the second study, 100 older palliative non-cancer patients participated. Cancer patients were significantly younger than non-cancer patients (75.68 years versus 81.02 years, t (498) = 6.17, *p* < 0.001) and significantly fewer cancer patients (72.5%) had a geriatric risk profile compared to the non-cancer patients (90.0%) (*χ*^*2*^ (1) = 13.43; *p* < 0.001). To control for age and geriatric risk profile, 100 cancer patients were matched to the 100 non-cancer patients on these two variables. The matching variables had to be identical to allow two patients to be matched. If more than one patient could be matched with another patient, an ad random match was drawn using a random number list in SPSS. A match was found for all cases. Only those 200 patients were included in the analysis. No missing data were present.

The difference in symptoms between both groups was measured using an independent sample t-test for continuous variables, Wilcoxon Mann-Whitney U-test for ordinal variables and Chi-square test for nominal variables.

A *p*-value < 0.05 was considered statistically significant. Data were analysed using SPSS, version 22 (Chicago, IL, USA).

## Results

### Demographics

A total of 200 patients were included in the analysis (Table 1). Of these, 100 patients had a primary diagnosis of cancer, and the remaining patients had a primary diagnosis of heart failure (22.5%), obstructive lung disease (13.5%), renal failure (12.5%), or liver failure (1.5%). Approximately 49.5% were men, and 50.5% were women. The mean age of the patients was 81.1 years (SD 7.8). Most patients had a geriatric risk profile (87.5%). More cancer patients (53.0%) were married and still lived with their partner as opposed to the non-cancer patients (39.0%). There were no significant differences between the cancer and non-cancer patients in demographic or clinical characteristics.

**Table 1:** Demographic and Clinical Characteristics of Patients (*N* = 200)

	Total sample (*N* = 200)		Cancer patients (*N* = 100)		Non-cancer patients (*N* = 100)	
	Mean (SD)	N (%)	Mean (SD)	N (%)	Mean (SD)	N (%)
Age (years)	81.1 (7.8)		81.12 (7.5)		81.02 (8.0)	
Gender						
Male		99 (49.5)		49 (49.0)		50 (50.0)
Female		101 (50.5)		51 (51.0)		50 (50.0)
Living status						
Married		92 (46.0)		53 (53.0)		39 (39.0)
Widow/widower		81 (40.5)		35 (35.0)		46 (46.0)
Unmarried		27 (13.5)		12 (12.0)		15 (15.0)
Living arrangements						
Living alone		76 (38.0)		35 (35.0)		41 (41.0)
Living with partner		94 (47.0)		53 (53.0)		41 (41.0)
Living with others		30 (15.0)		12 (12.0)		18 (18.0)
Pathology						
Cancer		100 (50.0)		100 (100.0)		
Heart failure		45 (22.5)				45 (45.0)
Obstructive lung disease		27 (13.5)				27 (27.0)
Renal failure		25 (12.5)				25 (25.0)
Liver failure		3 (1.5)				3 (3.0)
Type of admitted ward						
Internal ward		154 (77.0)		82 (82.0)		72 (72.0)
Geriatric ward		46 (23.0)		18 (18.0)		28 (28.0)
Number of comorbidities	2.1 (1.6)		2.2 (1.6)		2.1 (1.6)	
fTRST^a^						
No risk profile		25 (12.4)		15 (15.0)		10 (10.0)
Geriatric risk profile		175 (87.5)		85 (85.0)		90 (90.0)

No statistical differences between the groups were observed using the independent sample t-test for continuous variables and the Chi-square test for nominal variables. SD: Standard Deviation; N: number of patients. ^a^fTRST: Flemish Triage Risk Screening Tool. A score of ≥ 2 represents a geriatric risk profile, while a score of 0 and 1 is considered to be normal

### Difference in number of symptoms

On average, patients reported 17.28 symptoms (SD 5.05; range 6–31) (Table 2). Cancer patients reported significantly fewer symptoms than non-cancer patients (*t* (198) = 2.72; *p* = 0.007). Cancer patients reported significantly fewer physical symptoms (Fig. 1) than non-cancer patients (*t* (198) = 2.49; *p* = 0.013). Significantly fewer cancer patients (79.0%) experienced functional dependence compared to non-cancer patients (90.0%) (*χ*^*2*^ (1) = 4.62; *p* = 0.032). There was no significant difference between the two groups for the number of psychological symptoms.

**Table 2:** Number of Symptoms per Patient (*N* = 200)

	Total sample (*N* = 200)	Cancer patients (*N* = 100)	Non-cancer patients (*N* = 100)	t	df	*p*-value
	Mean (SD)	Mean (SD)	Mean (SD)			
All symptoms^a^	17.28 (5.05)	16.32 (4.80)	18.23 (5.13)	2.72	198	**0.007**
Physical symptoms^a^	9.14 (3.22)	8.58 (3.05)	9.70 (3.30)	2.49	198	**0.013**
Psychological symptoms^a^	3.70 (2.48)	3.73 (2.47)	3.66 (2.44)	−0.20	198	**0.840**
Functional dependence^b^	169 (84.5)	79 (79.0)	90 (90.0)	4.62	1	**0.032**

^a^The maximal number of symptoms was 40. The maximal number of physical symptoms was 24. The maximal number of psychological symptoms was 10. ^b^Functional dependence defined as experiencing difficulties on at least one functional item (self-care, moving inside, and moving outside) was a dichotomous variable. Therefore numbers with percentages instead of means with SD are reported. The difference between groups was assessed using the Chi-square test

### Difference in frequency of symptoms

The frequency of symptoms in both patient groups is displayed in Figure 1. The five symptoms with the highest frequency in cancer patients were dry mouth, physical fatigue, difficulties moving outside, difficulties with self-care, and lack of energy. The five symptoms with the highest frequency in non-cancer patients were difficulties moving outside, difficulties with self-care, lack of energy, difficulties moving inside, and shortness of breath. The differences in the symptom frequency between cancer and non-cancer patients are presented in Table 3. Cancer patients reported significantly higher frequencies compared to non-cancer patients for physical fatigue (*Z* = − 2.78; *p* = 0.005), psychological pain (*Z* = − 2.16; *p* = 0.031), and not being satisfied with social life (*Z* = − 3.46; *p* = 0.001). Non-cancer patients reported significantly higher frequencies of the following symptoms compared to cancer patients: shortness of breath (*Z* = − 2.16; *p* = 0.031), itching (*Z* = − 2.07; *p* = 0.038), airway mucus (*Z* = − 2.01; *p* = 0.045), confusion (*Z* = − 2.46; *p* = 0.014), lack of willpower (*Z* = − 5.01; *p* < 0.001), difficulties with self-care (*Z* = − 2.00; *p* = 0.045), difficulties moving inside (*Z* = − 4.64; p < 0.001), and difficulties moving outside (*Z* = − 2.71; *p* = 0.007).

**Table 3:** Difference in Symptom Frequency and Intensity between Cancer and Non-Cancer Patients

Frequency							Intensity							
	Cancer patients (*N* = 100)		Non-Cancer patients (*N* = 100)		Wilcoxon Mann Whitney U-test		Cancer patients			Non-Cancer patients			Wilcoxon Mann Whitney U-test	
	Median(25–75 IQ)	Mean(SD)	Median(25–75 IQ)	Mean(SD)	Z	*p*-value	N	Median(25–75 IQ)	Mean(SD)	N	Median(25–75 IQ)	Mean(SD)	Z	*p*-value
Physical														
Lack of appetite	2.0 (0.0–4.0)	1.89 (1.69)	2.0 (0.0–3.0)	1.48 (1.46)	−1.74	0.082	63	2.0 (0.0–3.0)	1.83 (1.28)	58	2.0 (1.0–3.0)	1.84 (0.97)	−0.40	0.692
Fatigue (physical)	3.0 (2.0–4.0)	2.53 (1.55)	2.0 (2.0–3.0)	2.30 (1.40)	−1.53	0.127	79	2.0 (2.0–3.0)	2.47 (0.92)	79	2.0 (2.0–3.0)	2.47 (0.70)	−0.87	0.387
Pain (physical)	2.0 (0.0–3.0)	1.66 (1.65)	2.0 (0.0–4.0)	1.98 (1.68)	−1.39	0.164	55	3.0 (2.0–3.0)	2.40 (0.76)	64	3.0 (2.3–3.0)	2.69 (0.61)	−2.30	**0.021**
Lack of energy	3.0 (0.0–4.0)	2.26 (1.69)	3.0 (2.0–4.0)	2.55 (1.49)	−0.98	0.325	68	3.0 (2.0–3.0)	2.43 (0.98)	81	3.0 (3.0–3.0)	2.70 (0.58)	−1.47	0.141
Nausea	0.0 (0.0–1.0)	0.59 (1.01)	0.0 (0.0–1.0)	0.53 (0.96)	−0.19	0.851	27	3.0 (2.0–3.0)	2.41 (0.93)	27	3.0 (2.0–3.0)	2.30 (0.99)	−0.39	0.699
Sleeplessness	0.0 (0.0–3.0)	1.23 (1.53)	2.0 (0.0–3.0)	1.61 (1.56)	−1.87	0.062	44	3.0 (1.3–3.0)	2.18 (1.02)	60	3.0 (2.0–3.0)	2.25 (0.97)	−0.28	0.781
Vomiting	0.0 (0.0–0.0)	0.32 (0.82)	0.0 (0.0–0.0)	0.42 (0.90)	−0.76	0.448	15	3.0 (2.0–3.0)	2.47 (0.92)	20	3.0 (2.3–3.0)	2.70 (0.57)	−0.65	0.514
Dizziness	0.0 (0.0–2.0)	0.78 (1.12)	0.0 (0.0–2.0)	0.76 (1.09)	−0.05	0.643	36	2.5 (2.0–3.0)	2.17 (1.03)	37	2.0 (2.0–3.0)	2.14 (0.86)	−0.55	0.586
Heartburn/belching	0.0 (0.0–0.0)	0.42 (0.93)	0.0 (0.0–0.0)	0.39 (0.93)	−0.46	0.641	21	2.0 (1.0–3.0)	1.90 (1.04)	18	2.0 (1.0–3.0)	1.94 (0.80)	−0.13	0.894
Tingling hand/ft	0.0 (0.0–2.0)	1.10 (1.61)	0.0 (0.0–2.0)	1.11 (1.56)	−0.15	0.883	36	2.0 (1.0–3.0)	2.08 (1.05)	38	3.0 (2.0–3.0)	2.45 (0.83)	−1.59	0.111
Difficulties concentrating	0.0 (0.0–2.0)	0.98 (1.48)	0.0 (0.0–2.0)	0.81 (1.13)	−0.16	0.874	35	2.0 (1.0–3.0)	2.00 (1.11)	39	3.0 (2.0–3.0)	2.46 (0.85)	−1.88	0.061
Sore mouth/pain when swallowing	0.0 (0.0–0.0)	0.50 (1.20)	0.0 (0.0–0.0)	0.53 (1.11)	−0.57	0.566	17	3.0 (2.0–3.0)	2.47 (0.72)	21	3.0 (2.0–3.0)	2.38 (0.92)	−0.05	0.960
Shortness of breath	2.0 (0.0–3.0)	1.59 (1.55)	2.0 (1.0–4.0)	2.34 (1.49)	−3.42	**0.001**	56	2.0 (2.0–3.0)	2.11 (1.00)	81	3.0 (3.0–3.0)	2.18 (0.95)	−4.15	**< 0.001**
Dry mouth	3.0 (2.0–4.0)	2.58 (1.35)	2.0 (2.0–3.0)	2.25 (1.36)	−1.85	0.065	85	2.0 (0.5–3.0)	1.86 (1.23)	80	2.5 (1.0–3.0)	2.44 (0.64)	−1.49	0.138
Cough	0.5 (0.0–2.0)	1.09 (1.25)	0.5 (0.0–2.0)	1.18 (1.32)	−0.42	0.678	50	2.0 (0.8–3.0)	1.88 (1.22)	50	3.0 (2.0–3.0)	2.40 (0.89)	−1.94	0.052
Urinary incontinence	0.0 (0.0–2.0)	0.94 (1.28)	0.0 (0.0–2.0)	0.91 (1.50)	−0.81	0.420	40	3.0 (2.0–3.0)	2.40 (1.01)	30	3.0 (2.0–3.0)	2.47 (0.84)	−0.24	0.811
Faecal incontinence	0.0 (0.0–0.0)	0.29 (0.82)	0.0 (0.0–0.0)	0.48 (1.07)	−1.22	0.222	13	3.0 (3.0–3.0)	2.85 (0.38)	19	3.0 (2.0–3.0)	1.86 (1.16)	−1.22	0.225
Itching	0.0 (0.0–0.0)	0.38 (0.94)	0.0 (0.0–1.0)	0.62 (1.06)	−2.07	**0.038**	16	1.5 (0.3–3.0)	1.69 (1.30)	29	2.0 (1.0–3.0)	2.58 (0.56)	−0.45	0.650
Changes in food tasting	0.0 (0.0–2.5)	0.95 (1.67)	0.0 (0.0–2.0)	0.95 (1.54)	−0.47	0.636	25	3.0 (1.5–3.0)	2.36 (1.04)	31	3.0 (2.0–3.0)	2.48 (0.66)	−0.14	0.892
Airway mucus	0.0 (0.0–2.0)	1.07 (1.39)	2.0 (0.0–3.0)	1.46 (1.40)	−2.01	**0.045**	43	2.0 (0.0–3.0)	1.93 (1.24)	58	3.0 (2.0–3.0)	2.48 (0.66)	−1.82	0.069
Confusion	0.0 (0.0–0.0)	0.25 (0.73)	0.0 (0.0–0.8)	0.51 (0.93)	−2.46	**0.014**	11	2.0 (1.0–3.0)	2.09 (1.04)	25	3.0 (2.0–3.0)	2.48 (0.77)	−1.09	0.274
Constipation	0.0 (0.0–2.0)	1.22 (1.44)	2.0 (0.0–3.0)	1.47 (1.50)	−1.18	0.239	46	3.0 (2.0–3.0)	2.33 (1.01)	55	2.0 (1.0–3.0)	2.09 (0.98)	−1.44	0.150
Diarrhoea	0.0 (0.0–1.0)	0.63 (1.17)	0.0 (0.0–1.0)	0.60 (1.09)	−0.18	0.860	27	3.0 (1.0–3.0)	2.26 (1.10)	26	3.0 (2.0–3.0)	2.50 (0.91)	−0.69	0.492
Weight loss^a^	38 (38.0)	–	41 (41.0)	–	0.19	0.664	–	–	–	–	–	–	–	–
Psychological														
Fatigue (psychological)	2.0 (0.0–3.0)	1.54 (1.52)	0.0 (0.0–2.0)	0.96 (1.39)	−2.78	**0.005**	56	3.0 (2.3–3.0)	2.72 (0.57)	38	2.0 (2.0–3.0)	2.32 (0.66)	−3.10	**0.002**
Pain (psychological)	0.0 (0.0–2.0)	0.96 (1.37)	0.0 (0.0–0.0)	0.57 (1.14)	−2.16	0.031	36	3.0 (3.0–3.0)	2.72 (0.57)	23	3.0 (2.0–3.0)	2.43 (0.73)	−1.73	0.084
Brooding	2.0 (0.0–3.0)	1.49 (1.42)	2.0 (0.0–2.8)	1.48 (1.41)	−0.08	0.940	57	3.0 (2.0–3.0)	2.51 (0.74)	59	2.0 (1.0–3.0)	1.90 (0.90)	−3.83	**< 0.001**
Depressed mood	0.0 (0.0–2.0)	1.11 (1.24)	0.0 (0.0–2.0)	1.09 (1.33)	−0.29	0.769	49	3.0 (2.0–3.0)	2.55 (0.68)	46	2.0 (2.0–3.0)	2.30 (0.70)	−1.94	0.053
Lack of willpower	0.0 (0.0–2.0)	0.86 (1.22)	2.0 (0.0–3.0)	1.91 (1.51)	−5.01	**< 0.001**	37	2.0 (1.5–3.0)	1.97 (1.07)	70	2.0 (2.0–3.0)	2.63 (0.57)	−3.39	**0.001**
Nervousness	0.0 (0.0–2.0)	1.27 (1.52)	0.0 (0.0–2.0)	1.11 (1.31)	−0.55	0.582	48	2.0 (0.3–3.0)	1.90 (1.21)	48	2.0 (2.0–3.0)	2.00 (0.85)	−0.24	0.813
Tension	0.0 (0.0–2.0)	0.90 (1.31)	0.0 (0.0–2.0)	0.93 (1.20)	−0.47	0.641	36	3.0 (2.0–3.0)	2.39 (0.84)	42	2.0 (2.0–3.0)	2.12 (0.63)	−2.22	**0.026**
Anger	0.0 (0.0–0.0)	0.55 (1.04)	0.0 (0.0–0.8)	0.51 (0.96)	−0.03	0.978	24	3.0 (2.0–3.0)	2.50 (0.89)	25	2.0 (1.0–3.0)	1.84 (1.14)	−2.29	**0.022**
Loneliness	0.0 (0.0–2.0)	0.92 (1.26)	0.0 (0.0–2.0)	1.13 (1.45)	−0.94	0.347	39	3.0 (2.0–3.0)	2.36 (0.87)	44	3.0 (2.0–3.0)	2.39 (0.75)	−0.09	0.931
Fear	0.0 (0.0–1.0)	0.62 (1.14)	0.0 (0.0–0.0)	0.37 (0.85)	−1.63	0.104	27	3.0 (2.0–3.0)	2.41 (0.89)	18	2.0 (2.0–3.0)	2.28 (0.67)	−1.03	0.305
Functional														
Difficulties with self-care	3.5 (0.0–4.0)	2.32 (1.87)	4.0 (2.0–4.0)	2.89 (1.56)	−2.00	**0.045**	64	1.0 (0.0–2.8)	1.23 (1.27)	82	2.0 (1.0–3.0)	1.67 (1.03)	−2.34	**0.019**
Difficulties with moving inside	4.0 (0.0–4.0)	1.35 (1.82)	3.0 (1.0–4.0)	2.45 (1.71)	−4.64	**< 0.001**	37	3.0 (2.0–3.0)	2.46 (0.96)	77	3.0 (2.0–3.0)	2.38 (0.90)	−0.71	0.480
Difficulties with moving outside	4.0 (0.0–4.0)	2.40 (1.92)	4.0 (3.0–4.0)	3.23 (1.35)	−2.71	**0.007**	62	3.0 (1.0–3.0)	2.05 (1.25)	89	3.0 (2.0–3.0)	2.29 (0.96)	−0.87	0.384
Social														
Not feeling supported	0.0 (0.0–0.0)	0.40 (1.04)	0.0 (0.0–0.0)	0.33 (0.80)	−0.09	0.927	16	0.0 (0.0–0.0)	0.00 (0.00)	16	0.0 (0.0–0.8)	0.25 (0.45)	0.04	0.239
Not satisfied with social life	0.0 (0.0–2.0)	1.04 (1.48)	0.0 (0.0–0.0)	0.45 (1.00)	−3.46	**0.001**	42	0.0 (0.0–0.3)	0.26 (0.48)	20	0.0 (0.0–0.8)	0.30 (0.57)	−0.15	0.880
Existential														
Experiencing life as not meaningful	0.0 (0.0–2.0)	1.21 (1.54)	2.0 (0.0–2.0)	1.35 (1.22)	−1.28	0.201	46	0.0 (0.0–0.0)	0.15 (0.42)	53	0.0 (0.0–1.0)	0.53 (0.62)	−3.75	**< 0.001**

^a^Weight loss is a dichotomous variable. The number of patients reporting the symptom with its percentage are reported. Difference between groups was measured using the Chi-square test

### Differences in intensity of symptoms

The intensity of symptoms in both patient groups is displayed in Figure 1. The following five symptoms reported by at least 50% of the cancer patients received the highest intensity: psychological fatigue, brooding, physical fatigue, lack of energy, and psychical pain. The following five symptoms were reported by at least 50% of the non-cancer patients as being the highest intensity: lack of energy, physical pain, lack of willpower, airway mucus, and physical fatigue. The differences in intensity of symptom between cancer and non-cancer patients are presented in Table 3. Cancer patients reported significantly higher intensity levels than non-cancer patients for psychological fatigue (*Z* = − 3.10; *p* = 0.002), brooding (*Z* = − 3.83; *p* < 0.001), tension (*Z* = − 2.22; *p* = 0.026), and anger (*Z* = − 2.29; *p* = 0.022). Non-cancer patients reported significantly higher intensity levels compared to cancer patients for physical pain (*Z* = − 2.30; *p* = 0.021), shortness of breath (*Z* = − 4.15; *p* < 0.001), lack of willpower (*Z* = − 3.39; *p* = 0.001), difficulties with self-care (*Z* = − 2.34; *p* = 0.019), and perceiving life as not meaningful (*Z* = − 3.75; *p* < 0.001).

## Discussion

This study is the first to evaluate the differences in symptom patterns between older hospitalised palliative cancer and non-cancer patients. Overall, similarities in symptom patterns could be observed between cancer and non-cancer patients. Patients in both groups experienced problems with functionality, physical fatigue, lack of energy, shortness of breath and physical pain with a high frequency and physical fatigue, lack of energy, and physical pain with a high intensity. Similar symptom patterns were also observed in the systematic reviews of Solano et al. (2006) and Moens et al. (2014) concerning the prevalence of symptoms in palliative patients with different diseases [4, 5]. Although the ranking in frequency and intensity of symptoms was similar in both groups, some minor differences could be observed in the frequency and intensity of symptoms. Non-cancer patients experienced significantly more physical symptoms and functional dependence compared to cancer patients. In addition, the frequency and intensity of functional dependence were significantly higher for non-cancer patients compared to the cancer group. A possible explanation of this finding could be that interventions to decrease symptoms focus more on palliative cancer patients than on non-cancer patients. Only in recent years has research emphasised that more attention should be paid to palliative non-cancer patients [3, 4]. A few physical symptoms occurred more often in cancer patients such as physical fatigue and dry mouth. These symptoms are common side effects of chemotherapy.

Regarding psychological symptoms, no differences were observed between the groups in terms of the number of psychological symptoms per patient. However, when examining the individual symptoms, cancer patients appeared to experience a higher frequency and intensity of psychological symptoms, with the exception of lack of willpower. The high frequency and intensity of psychological symptoms might be related to patients’ illness perceptions, which help them make sense of their experienced symptoms and subsequently guide their coping strategies [16]. For patients without cancer, the long-lasting and fluctuating nature of chronic illness presents a challenge for identifying ways to cope with their illness [17]. In contrast, a cancer diagnosis is marked by an immediate change from well-being to feelings of uncertainty and the threat of death [18].

### Implications for clinical practice and research

The occurrence and intensity of symptoms were high in both older palliative cancer and non-cancer patients. It is worth noting that patients in the present study were not patients in a terminal phase, indicating that multiple symptoms with a high frequency and intensity are already experienced before the end-of-life stage. Accordingly, attention should be devoted to older patients’ experience of symptoms in the palliative phase to decrease the frequency and intensity of their symptoms. Healthcare professionals should be sensitised to this high symptom occurrence in both cancer and non-cancer patients. In addition, they should be educated on systematic symptom assessment in multiple domains.

Additionally, the commonalities in symptom patterns indicate that both cancer and non-cancer patients may need support in reducing symptom burden. A referral to palliative care should therefore be based on the needs of the individual patient, rather than diagnosis-based. This was also emphasised by Moens et al. [4] and Solano et al. [5] in their review. Both patient groups might benefit from a referral to palliative support teams. These teams are specialised in managing symptoms to improve quality of life, and the literature indicates that an early referral to palliative care increases patients’ quality of life [19]. For clinicians, it is difficult to identify when patients should be referred to palliative care [3]. This is especially true for non-cancer patients because of the unpredictable course of their illness [3]. Despite this challenge, healthcare professions should be educated about referring patients to palliative care early on in their care which should be based on the patient’s needs. Moreover, to improve this referral, attention should be paid to patient, and professional-related barriers to referring patients to palliative care, such as a lack of knowledge and an inadequate perception of palliative care [3]. Also, a referral to a specialised geriatric care team might be beneficial for patients who are confronted with age-related disability, chronic conditions, decreased functionality and psychosocial problems and the care goal is to increase the patient’s independence, physical and mental health and functionally [20, 21]. Healthcare professionals could use a screening tool in older patients to identify those patients in need of a further geriatric evaluation [22]. Nevertheless, the benefits of referral should be weighed against the risk of fragmentation of care when involving different healthcare professionals.

The observed differences between cancer and non-cancer patients indicate that there are some disease-specific differences that should be addressed in clinical practice. Healthcare professionals should devote attention to both generic symptoms and disease-specific symptoms. Education on these aspects is advised.

In the present study, cancer and non-cancer patients were compared. Further research could evaluate the differences in symptoms between different non-cancer patient groups. This was not possible in the present study due to small number of patients in the subgroups of non-cancer patients (range 3–45).

### Strengths and limitations

This study has some strengths and limitations. To our knowledge, this study is the first to evaluate the differences in symptoms between older hospitalised palliative cancer and non-cancer patients using the same instrument and time point to evaluate symptoms. This consistency enhanced the validity of the results. A limitation of the present study is the possible difference between groups in terms of additional, non-collected demographic and clinical variables. Nevertheless, both groups were matched for age and geriatric risk profile to decrease a possible influence of these factors on the outcomes. The matched cancer patients were older and had a higher geriatric risk profile compared to the original sample. Consequently, the cancer group might be less representative for the larger population. Also, the difference in the provision of palliative care between the two groups was not evaluated. Nevertheless, patients were recruited from the same hospitals and the difference in symptoms was minimal which might imply that the palliative care provision was rather similar between the two patient groups. Another limitation was the possible risk of systematic and researcher bias in the data-collection because the data was collected by two researchers. To overcome this, the second researcher was instructed by the primary researcher.

## Conclusion

Similarities in symptom patterns could be observed between cancer and non-cancer patients. In both groups, a high frequency was observed for functionality, physical fatigue, lack of energy, shortness of breath, and physical pain, and a high intensity was observed for physical fatigue, lack of energy, and physical pain. Despite these similarities, non-cancer patients experienced significantly more physical symptoms and functional dependence compared to cancer patients, and cancer patients experienced a higher frequency and intensity of psychological symptoms. Nevertheless, the similarities were larger than the differences which indicates that a need-based referral to palliative care would be more beneficial than a diagnoses-based referral. Healthcare professionals should be sensitised to the high symptom occurrence in both cancer and non-cancer patients. Additionally, they should be educated about systematic symptom assessment in multiple domains by accounting for the occurrence of generic symptoms and disease-specific symptoms.

## Abbreviations

ASPE: Assessment symptoms palliative elderly
fTRST: Flemish triage risk screening tool
SD: Standard deviation
